# Photoconduction Properties in Tungsten Disulfide Nanostructures

**DOI:** 10.3390/nano13152190

**Published:** 2023-07-27

**Authors:** Hemanth Kumar Bangolla, Yueh-Chien Lee, Wei-Chu Shen, Rajesh Kumar Ulaganathan, Raman Sankar, He-Yun Du, Ruei-San Chen

**Affiliations:** 1 Graduate Institute of Applied Science and Technology, National Taiwan University of Science and Technology, Taipei 10607, Taiwan; hemanthbangolla@gmail.com; 2 Department of Electronic Engineering, Lunghwa University of Science and Technology, Taoyuan 33306, Taiwan; yclee8858@gmail.com; 3 Department of Electronic Engineering, National Taiwan University of Science and Technology, Taipei 10607, Taiwan; m10202337@mail.ntust.edu.tw; 4 Institute of Physics, Academia Sinica, Taipei 115201, Taiwan; urajeshkumariitr@gmail.com (R.K.U.); sankarndf@gmail.com (R.S.); 5 Department of Chemical Engineering, Ming Chi University of Technology, New Taipei City 24301, Taiwan

**Keywords:** tungsten disulfide, nanoflake, photoconductivity, photodetector, responsivity, normalized gain

## Abstract

We reported the photoconduction properties of tungsten disulfide (WS_2_) nanoflakes obtained by the mechanical exfoliation method. The photocurrent measurements were carried out using a 532 nm laser source with different illumination powers. The results reveal a linear dependence of photocurrent on the excitation power, and the photoresponsivity shows an independent behavior at higher light intensities (400–4000 Wm^−2^). The WS_2_ photodetector exhibits superior performance with responsivity in the range of 36–73 AW^−1^ and a normalized gain in the range of 3.5–7.3 10^−6^ cm^2^V^−1^ at a lower bias voltage of 1 V. The admirable photoresponse at different light intensities suggests that WS_2_ nanostructures are of potential as a building block for novel optoelectronic device applications.

## 1. Introduction

In the modern technology era, optoelectronic devices have been established as one of the most ambitious fields of study. Photodetectors are the sub-class of optoelectronic devices that can convert incident light into electrical signals precisely. Photodetectors are vital components to achieve devices with multi-functionality, and hence gained more attention in many applications such as imaging, optical communications, light sensing, and biomedical instruments [1,2,3]. Photodetectors can be divided into two categories based on detection mechanism, namely, photon or quantum detectors and thermal detectors. The photon detectors that include photoconductors, photodiodes, and photo-field effect transistors (photo-FETs) are widely studied due to the existence of band gaps and fast inter-band optical transition. The thermal detectors are either bolometers or thermopiles. Due to their indirect photoelectric conversion, thermal detectors have a relatively slow photoresponse speed [4]. A photoconductor is a fundamental photodetector that is simply a semiconductor channel with ohmic contacts on both ends that works on a photoconductive effect. The photoconductive effect is a process in which the conductivity of a semiconductor material increases due to photon absorption when illuminated by light energy larger than the bandgap of the semiconductor. A photoconductor possesses a gain that can be greater than unity. The high gain will reduce the response speed of the photoconductor. In order to achieve a photoconductor’s desired overall performance, a trade-off between gain and response speed must be made [5].

The advent of nanomaterials leads to improving the performance and shrinking the size of novel devices due to their exceptional properties governed by high surface-to-volume ratio and quantum effects at a nanoscale regime [3]. Recently, the transition metal dichalcogenides (TMDs) belonging to the two-dimensional (2D) family have been promoted as novel candidates for fabricating miniature electronic and optoelectronic devices for next-generation devices due to their excellent electrical and optical properties [6,7,8,9,10,11]. The 2D TMDs have a honeycomb molecular structure of MX_2_, where M is a transition metal atom and X is a chalcogen atom. In 2D TMDs, the strong covalent bonded layers are stacked via weak van der Wall interactions. Among the TMDs, the most extensive research has been done on molybdenum disulfide (MoS_2_). The first mono-layered MoS_2_ phototransistor exhibits a fast response time of 50 ms, but shows a low responsivity of 7 mAW^−1^ due to its poor carrier mobility and low optical absorbance [12]. The multi-layered MoS_2_ photodetectors show a responsivity in the range of 100–570 mAW^−1^ due to the increase of optical absorbance of multilayers [13,14].

The versatile compound tungsten disulfide (WS_2_), another promising member of the TMDs group, has been widely investigated in the field of optoelectronic device applications due to its high mobility and environmental stability [15,16,17,18,19]. The WS_2_ possesses an indirect bandgap (1.4 eV) in its bulk form, and it converts to a direct bandgap (2.1 eV) for a monolayer [20,21]. Moreover, WS_2_ has strong optical absorption, high spin-orbit coupling, and high photoluminescence and can be operated over wide temperatures [22,23,24]. The theoretical calculations suggest WS_2_ has a smaller electron-effective mass and thus has higher carrier mobility [25,26]. Each layer in the WS_2_ compound is composed of tungsten (W) atoms sandwiched between the sulfur (S) atoms (S-W-S). Hence, the WS_2_ bulk crystal consists of stacks of three atom sheets. It can be easily exfoliated into thin nanoflakes or nanosheets with strong in-plane covalent bonding and transferred onto an arbitrary substrate due to the weak van der Waals force between various sheets [27,28]. The exfoliated WS_2_ monolayers or multilayers attained exceptional significance in various applications such as photodetectors [22], field effect transistors [29], gas sensors [30], energy storage devices [31], light emitting diode elements [32], and catalysts [33]. The light absorption in monolayer TMDs is approximately 5–10% in the visible regime [34]. This is relatively higher than the conventional photodetector materials such as Si and GaAs in a comparable thickness [35]. However, the practical applications of monolayer TMDs have been restricted due to their thickness-limited absorption, bandgap-limited spectral response, and high Schottky barrier-limited charge collection efficiency [36]. In the WS_2_ monolayer, the conduction band (CB) edge is located at a higher energy than that of a MoS_2_ monolayer. This results in more severe issues in electrical contact as it forms higher Schottky barriers between WS_2_ and metal electrodes as compared to MoS_2_ [15]. Unlike monolayer TMDs, the thicker multilayer TMDs possess better electrical transport and higher light absorption coefficients [37,38]. Hence, the photodetectors based on multilayer TMDs can be achieved with high responsivity and a wide spectral regime [13,26].

The 2D WS_2_ material can be synthesized by both top-down and bottom-up approaches. The widely used top-down techniques are mechanical exfoliation, chemical/liquid exfoliation, and laser or electron irradiation. The bottom-up techniques that include chemical vapor deposition (CVD), atomic layer deposition (ALD), hydrothermal or electrochemical process, and molecular beam epitaxy (MBE) have been extensively studied [39]. In general, the synthesis technique should be simple, affordable, and scalable without the need for expensive machinery for low-cost production. Among the above-mentioned techniques, the mechanical exfoliation method is simple and does not require any sophisticated instruments. The exfoliation also produces nanoflakes with high crystalline quality [38]. In the exfoliation technique, the nanoflakes were peeled from the bulk crystals using scotch tape. A few reports are available on the photoresponse behavior of mechanically exfoliated 2D WS_2_ [3]. Lee et al. reported FET based on multi-layer WS_2_ with a thickness of ~20 nm and a photoresponsivity of ~0.27 A/W [40]. Huo et al. reported multilayered WS_2_ nanoflakes-based FET with a photoresponsivity of 5.7 A/W [30]. Huo et al. also reported a transistor based on a multi-layer MoS_2_–WS_2_ heterostructure. The planar device exhibits a photoresponsivity of 1.42 A/W [41].

In this work, the WS_2_ nanoflakes are exfoliated from the chemical vapor transport (CVT) grown crystals using a conventional mechanical exfoliation technique. For the fabrication of a photoconductor-type photodetector, the platinum (Pt) electrodes were deposited on a WS_2_ nanoflake using the focused ion beam (FIB) technique. The photoconduction properties of the device were investigated under the laser wavelength of 532 nm with different powers. The fabricated device shows good performance at a lower bias voltage of 1 V. The photodetector parameters such as responsivity, gain, and normalized gain were estimated and discussed.

## 2. Materials and Methods

### 2.1. WS_2_ Crystal Growth

Single crystals of WS_2_ were grown by the CVT method using the fine powders of sulfur (99.99%) and tungsten (99.99%) with the help of iodine (I_2_) as a transporting agent. At first, sulfur and tungsten powders were mixed with I_2_ and transferred into the quartz ampoule with a length of 30 cm. The inner and outer diameter of the quartz ampoule is 1.3 and 1.6 cm, respectively. Later, the quartz ampoule was evacuated to 10^−5^ Torr and sealed at one end. Next, the sealed ampoule was kept in the two-zone horizontal furnace maintained at temperatures of 1020 and 960 °C. The precursor powder was kept at the higher temperature of 1020 °C zone; once the powder started to melt and vaporize, the I_2_ transported the vaporized precursor to the other end of the tube; the temperature was maintained at 960 °C. After ten days of the process, the vaporized precursors were deposited as single crystals of 1–2 cm in length.

### 2.2. Fabrication of WS_2_ Photodetector

The WS_2_ nanoflakes were exfoliated from the bulk crystal using a conventional mechanical exfoliation technique using dicing tape. For the fabrication of a photoconductor-type photodetector, the WS_2_ nanoflakes were transferred onto a SiO_2_ (300 nm)/n^+^-Si substrate with pre-patterned Ti/Au electrodes. Next, two Pt metal contacts with a thickness of 100 nm were deposited on WS_2_ nanoflakes using the FIB technique. Finally, the electrical wires were connected to the Ti/Au electrodes using a silver paste to characterize the fabricated device. The Ti/Au electrodes are the interconnection between the Pt microelectrode and the millimeter-sized bonded wire.

### 2.3. Measurements and Characterization

The X-ray diffraction (XRD) pattern was measured using a D2 Phaser X-ray diffractometer, and the Raman spectroscopy was measured with an excitation wavelength of 532 nm using a Raman microscope (Renishaw InVia, Wotton-under-Edge, UK); these measurements were used to confirm the crystal structure of CVT-grown WS_2_ crystals. The height profiles were carried out to find the thickness of nanoflakes using atomic force microscopy (AFM, Bruker-ICON2-SYS, Billerica, MA, USA). Scanning electron microscopy (SEM, Hitachi S3000H, Tokyo, Japan) was used to capture the image of the nanoflake device to obtain the dimensions of the conduction channel. Focused ion beam (FIB, FEI Quanta 3D FEG) was utilized for the deposition of Pt contacts. The dark current-voltage (i_d_-V) curves and photoconductive measurements of the photodetector were carried out in a four-point probe electrical measurement system using Keithly 4200-SCS. A 532 nm laser source was used for illumination and the incident laser power was measured using a calibrated power meter (Ophir Nova II) with a silicon photodiode head (Ohir PD300-UV). A holographic diffuser was utilized to minimize the error in the power density calculation by broadening the laser beam size (~20 mm^2^).

## 3. Results and Discussion

### 3.1. WS_2_ Crystal Characterization

**Figure 1:**
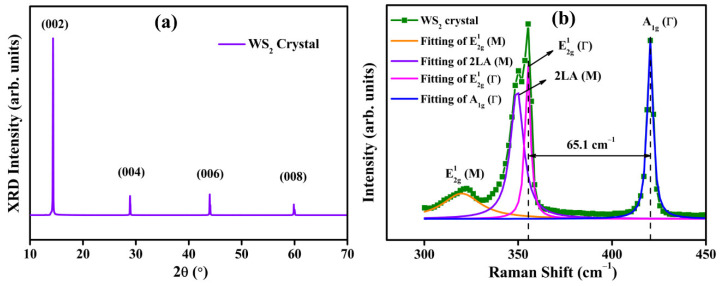
Structural characterization of CVT-grown WS_2_ bulk crystal. (**a**) X-ray diffraction pattern and (**b**) Raman spectrum.

The XRD pattern of CVT-grown WS_2_ crystal is shown in Figure 1a. The observed diffraction peaks at 2*θ* values of 14.3, 28.9, 43.9, and 59.8° are assigned to the (002), (004), (006), and (008) planes, respectively. The positions of sharp Bragg reflections confirm the 2H phase of WS_2_ crystals according to JCPDS card no. 08-0237 [42,43]. The 2H WS_2_ crystal lattice belongs to the P6_3_/mmc ($D_{6h}^{4}$) hexagonal space group that has space inversion symmetry [44]. The observed sharp and narrow peaks are an indication of the high crystal quality of WS_2_ crystals grown by the CVT technique. All diffraction peaks along the (00l) direction denote that the crystal growth is along the *c*-axis and the major preferential orientation is along the (002) plane. The absence of any binary or impurity phases in the XRD pattern demonstrates the exceptional quality of the CVT-grown crystals.

Figure 1b depicts the Raman spectrum of CVT-grown WS_2_ layered crystal. The multi-peak Lorentzian fitting is used for the individual peaks fitting and also for the deconvolution of a broad peak obtained at around 350 cm^−1^, which clearly separates the individual peaks from the overlapping. The observed Raman peaks at 319.9, 349.3, 355.1, and 420.2 cm^−1^ are attributed to $E_{2g}^{1}$ (M), 2LA (M), $E_{2g}^{1}$ (Γ), and $A_{1g}$ (Γ) modes of WS_2_ crystal, respectively [45,46,47]. The first-ordered dominant modes $E_{2g}^{1}$ (Γ) and $A_{1g}$ (Γ) are most commonly observed for 2H WS_2_ crystals [43,44,48]. The $E_{2g}^{1}$ mode is due to the in-plane vibrations of tungsten and sulfur atoms in the opposite direction, and the $A_{1g}$ mode is due to the out-of-plane vibrations in sulfur atoms. The separation between these two modes is 65.1 cm^−1^, which is consistent with the bulk WS_2_, and the separation reduces gradually with the decrease of the number of layers [48,49]. The second-order longitudinal acoustic mode 2LA (M) is very close to the $E_{2g}^{1}$ (Γ) and sometimes it overlaps the $E_{2g}^{1}$ (Γ) mode [45]. The full-width half maxima of 2LA, $E_{2g}^{1}$, and $A_{1g}$ modes are 8.3, 3.4, and 3.7 cm^−1^, respectively, and it denotes the high crystallinity of WS_2_ crystals grown by the CVT technique.

### 3.2. WS_2_ Nanoflake Device Characterization

**Figure 2:**
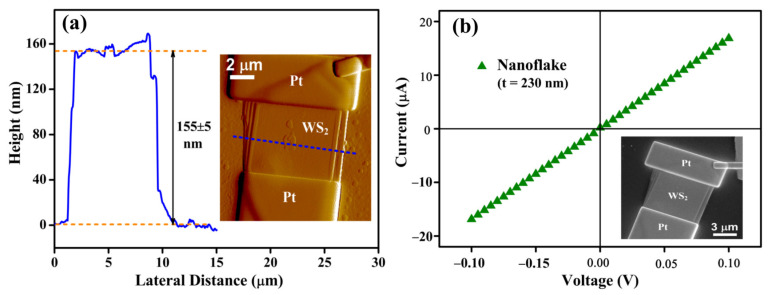
(**a**) AFM height profile of a WS_2_ nanoflake with a thickness of 155 nm; inset shows the AFM image of the respective device. (**b**) i_d_−V curve of a typical WS_2_ nanoflake with a thickness of 230 nm; inset shows SEM image of the WS_2_ nanoflake device of thickness 155 nm.

The thickness of the WS_2_ nanoflakes was calculated using the AFM height profile measurement as shown in Figure 2a. The thickness of a typical nanoflake is 155 ± 5 nm. The inset of Figure 2a shows the AFM picture of the WS_2_ nanoflake device with Pt contacts. The blue dotted line across the device denotes the position of the height profile measurement. Figure 2b depicts the i_d_-V characteristics of a typical WS_2_ nanoflake device in the range of −0.1 to +0.1 V. The linear i_d_-V curve confirms the ohmic contact between the WS_2_ nanoflake and FIB-deposited Pt contacts. The inset of Figure 2b represents the SEM image of the WS_2_ nanoflake device that is used to calculate the dimensions of the device. The conductivity (*σ*) of the WS_2_ nanoflake with a thickness of 230 nm was calculated using the relation [50,51]

(1) $$\sigma =G\frac{l}{A}=G\frac{l}{wt}$$

where *G* is the electrical conductance and *l*, *w*, and *t* are the length, width, and thickness of the conduction channel. *G* is given by *I*/*V*, which is obtained from the slope of i_d_-V curve, and the value is 1.69 × 10^−4^ Ω^−1^. The *l* and *w* are 4.22 μm and 2.65 μm, respectively, obtained from the SEM image of the nanoflake device with a thickness of 230 nm. The calculated conductivity of a typical nanoflake is 12 Ω^−1^cm^−1^.

### 3.3. Photoconduction Properties of WS_2_ Nanoflake

**Figure 3:**
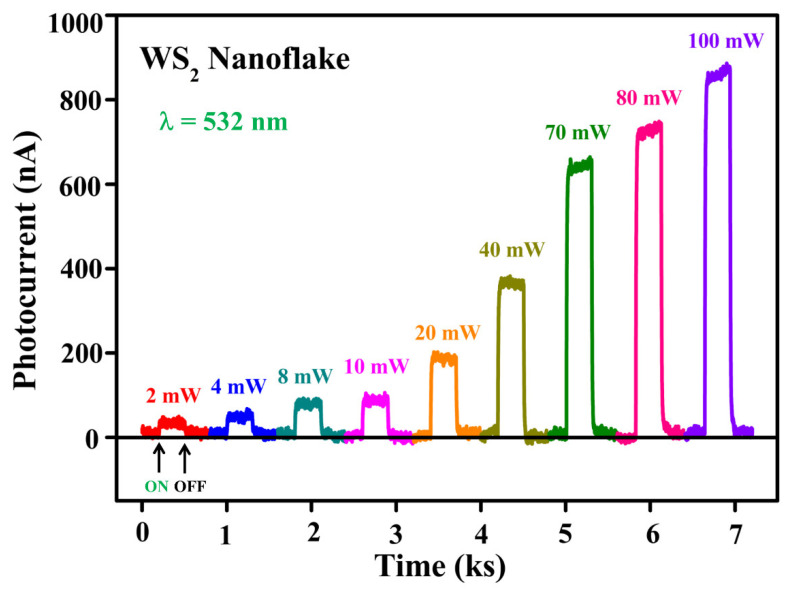
Photocurrent response of a WS_2_ nanoflake under laser illumination of a wavelength of 532 nm. The photocurrent is measured as a function of time under various powers at a fixed bias voltage of 1 V. The ON/OFF denotes the laser light condition.

Figure 3 depicts the photoresponse of a WS_2_ nanoflake with a thickness of 25 nm modulated by light power at an excitation wavelength of 532 nm. The photocurrent was measured for different light powers varying from 2 to 100 mW. A constant biasing voltage of 1 V was applied for the measurement of photocurrent as a function of time. First, we have recorded one cycle of photocurrent response for each light power separately. Next, the photocurrent measurements of different powers were combined to clearly present the change in photocurrent with respect to different powers. The ON and OFF states denote the laser light conditions for single light power. When the laser was turned on, the photocurrent increased quickly, and we waited until it saturated. Once it reached saturation, the laser was turned off, and the photocurrent was dropped immediately and then reached saturation. The background dark current was subtracted from photoresponse curves to represent the photocurrent curves. It is clear from the photoresponse curves that the photocurrent increases with the increase of light power. Generally, a large number of photons of high light intensity create a higher number of electron-hole pairs, and thus the photocurrent increases. The periodic nature of the photoresponse curve under different light powers is an indication of good stability and reproducibility of fabricated WS_2_ devices. With the increase of light power up to 100 mW, we did not observe any photocurrent saturation in the WS_2_ nanoflake, and hence the WS_2_ photodetectors can be suitable for operation in the linear region.

**Figure 4:**
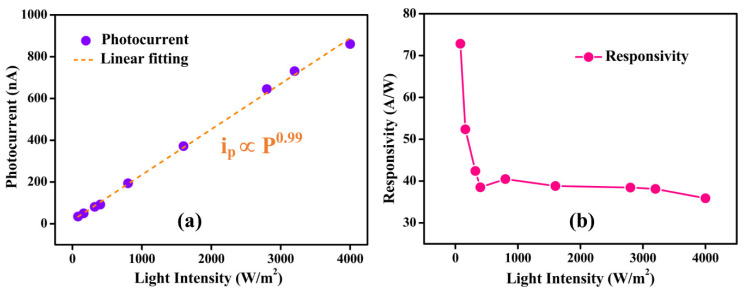
The dependence of (**a**) photocurrent and (**b**) responsivity on incident light intensities from 80 to 4000 Wm^−2^. The photocurrent data points were fitted using linear function.

To interpret the significant dependence of the photocurrent on the illumination intensity, the plot drawn between the photocurrent and light intensity in the range of 80–4000 Wm^−2^ is shown in Figure 4a. The photocurrent strongly depends on the light intensity, and the experimental data can be fitted using a power law given by $i_{p}=aP^{\beta }$, where *i_p_* is the photocurrent, *a* is the scaling constant, *P* is the light power, and *β* is an exponent [52]. The power law is well-fitted to the experimental data with *β* = 0.99. Generally, *β* values are in the range of 0 to 1. The deviation of the *β* value from unity is the indication of the presence of complex processes such as generation, trapping, and recombination of electron-hole within the semiconductor [53]. In our case, the *β* value is near unity, which indicates that the exfoliated WS_2_ nanoflake was of high quality with very few defects [54].

The photodetectors were characterized by several crucial parameters such as responsivity (*R*), gain (*Γ*), and normalized gain ($\Gamma_{n}$) to evaluate their performance. The *R* is one of the most important figure-of-merits, which is a measure of the photodetector’s electrical response to the incident light and is obtained from the formula [7]

(2) $$R=\frac{i_{p}}{P}$$

where, *i_p_* is the photocurrent and *P* is the laser power incident on the projected area (*A*) of a photodetector, and it is given by P=IA=Iwl, where *I* is the light intensity and *w* and *l* are the width and length of the conducting channel, respectively [55]. The *R* values as a function of light intensity are shown in Figure 4b. We noted that R is sensitive to the lower light intensity (80–320 Wm^−2^) and insensitive to the higher light intensities (400–4000 Wm^−2^). R decreases with the increase of light intensity from 80 to 400 Wm^−2^, and a further increase of light intensity up to 4000 Wm^−2^ results in an almost constant R value. A similar dependency of *R* on light intensity was observed in WS_2_ monolayer [56] and SnS/rGO [57] photodetectors. The calculated *R* values are in the range of 36–73 AW^−1^, and this high responsivity may be due to the efficient absorption and optimized WS_2_ nanoflake device configuration. These values are higher than the other photodetectors based on 2D materials such as NbSe_2_ nanoflakes (*R*~2.3–3.8 AW^−1^) [50], MoS_2_ nanoflakes (*R*~20–30 AW^−1^) [58], and NbS_2_ nanoflakes (*R*~0.6 AW^−1^) [59]. The largest *R* value (73 AW^−1^) at lower light intensity (80 Wm^−2^) is owed to the weak recombination of photo-excited carriers [60].

Gain (Γ) is another figure-of-merit of photodetectors that determine the circulating number of photo carriers moving through a photoconductor per unit time before recombination. It is given by the ratio of the carrier lifetime (τ) to the transit time ($\tau_{t}$) between the electrodes [50,61].

(3) $$\Gamma =\frac{\tau }{\tau_{t}}=\frac{V}{l^{2}}\tau \mu$$

where, *l* is the electrodes inter distance, *μ* is the mobility, and *V* is the applied voltage. *Γ* linearly depends on *R* and it can be calculated using the formula [62,63]

(4) $$\Gamma =\frac{R}{\eta }\frac{h\nu }{q}$$

where, η is the external quantum efficiency, *q* is the charge of an electron, *h* is Planck’s constant, and ν is the frequency of the photon. The η value was calculated using the formula $\eta =1-e^{-\alpha t}$, where α is the absorption coefficient at the wavelength of 532 nm (2.33 eV) and *t* is the thickness of the nanoflake [50,58]. The reflection loss was eliminated to simply the η calculation. The α value of WS_2_ bulk is ~2 × 10^5^ cm^−1^ at a photon energy of 2.33 eV [64,65]. By considering the nanoflake thickness of 25 nm, the calculated η value is 0.39 (39%).

**Figure 5:**
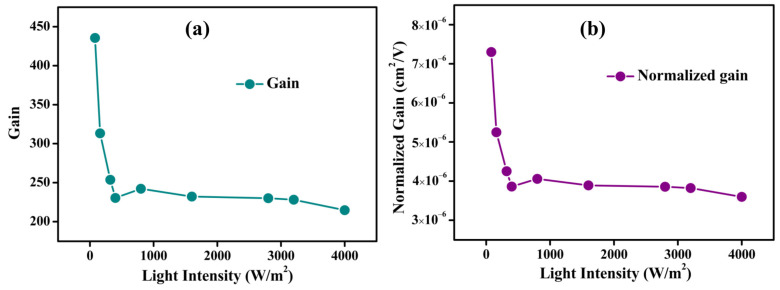
Variation of (**a**) gain and (**b**) normalized gain of a WS_2_ nanoflake photodetector with a variation of light intensity from 80 to 4000 Wm^−2^.

The determined *Γ* values as a function of light intensity are shown in Figure 5a. The *Γ* values follow a similar trend to *R*. The obtained *Γ* values are in the range of 215–436, with variation of light intensity from 80 to 4000 Wm^−2^. The *Γ* decreases with the increase of light intensity up to 400 Wm^−2^, and a further increase of light intensity up to 4000 Wm^−2^ leads to saturation in the *Γ* values. This may be attributed to the continuous filling of trap states upon illumination. Suppose the trap states are filled completely at a certain intensity of light, the excess electrons created by the higher light intensity cannot be trapped and thus decrease the average carrier lifetime. Hence, the photoconductive gain was reduced [4]. The *Γ* values (215–436) of our photodetector are superior to the reported photodetectors based on the MoS_2_ nanoflake (*Γ*~66–103) [58], WS_2_/Au NPs (*Γ*~30) [66], and the phototransistors based on MoS_2_ (*Γ*~0.2) and MoSe_2_ (*Γ*~5 × 10^−4^) [67].

Normalized gain ($\Gamma_{n}$) is independent of device geometry and can be considered a fair figure-of-merit to compare the performance of other devices. The photoconduction process is mainly involving the light absorption and movement of carriers between the electrodes. Numerous factors including carrier mobility, lifetime, applied bias, distance between electrodes, and efficiency of light absorption may affect the performance of photodetectors. $\Gamma_{n}$ is a measure of the intrinsic photoconductivity of the device and is given by the product of *η*, *τ*, and *µ* [61,68].

(5) $$\Gamma_{n}=\eta \tau \mu =\eta \frac{\Gamma }{\left(V/l^{2}\right)}=\frac{E}{q}\frac{l^{2}}{V}R$$

Figure 5b depicts the $\Gamma_{n}$ values as a function of light intensity. The $\Gamma_{n}$ values follow the *Γ* trend and the calculated values are in the range of 3.5–7.3 × 10^−6^ cm^2^V^−1^. The obtained values are higher than the AuNRs/MoS_2_/graphene device ($\Gamma_{n}$~8.63 × 10^−7^ cm^2^V^−1^) [68] but lower than the MoS_2_-UCNP nanocomposite ($\Gamma_{n}$~1.48 × 10^−4^ cm^2^V^−1^) [61] and InSe ($\Gamma_{n}$~3.2 cm^2^V^−1^) [55] based photodetectors. The moderate $\Gamma_{n}$ values of our device required further investigation of other parameters such as carrier lifetime and mobility.

The photodetector parameters *Γ* and $\Gamma_{n}$ were rarely investigated for the 2D material photodetectors. Hence, the comparison of devices based on *Γ* and $\Gamma_{n}$ becomes tough. We have compared our device performance with other reported WS_2_ photodetectors fabricated by different methods based on their operation region of wavelength, biasing voltage, and responsivity as summarized in Table 1. It is noticed that the WS_2_ nanoflake device obtained in the present work shows better responsivity at a lower bias voltage of 1 V. The high responsivity of our device also outperformed some CVD-grown WS_2_ monolayer-based photodetectors, and hence the WS_2_ nanoflakes can be a potential candidate for fabricating novel optoelectronic devices.

## 4. Conclusions

We successfully fabricated a visible photodetector using exfoliated WS_2_ nanoflakes and explored its photoconduction properties. The photocurrent increases with the increase of light intensity from 80 to 4000 Wm^−2^ and is well-fitted to the power law with an exponent value of 0.99. The photoresponsivity decreases with the increase of light intensity from 80 to 400 Wm^−2^, and a further increase of light intensity up to 4000 Wm^−2^ results in an almost constant R value. The fabricated device showed a stable photoresponse with some reproducible characteristics. The device exhibited good responsivity in the range of 36–73 AW^−1^, and the normalized gain was in the range of 3.5–7.3 × 10^−6^ cm^2^V^−1^ at a lower biasing voltage of 1 V. The obtained excellent photodetector parameters suggest a promising application of WS_2_ nanoflakes in future novel optoelectronic devices.

## Figures and Tables

**Table 1:** Comparison of WS_2_ photodetectors based on responsivity, fabrication method, and their operational wavelength with bias voltage.

Material	Fabrication Method	Wavelength (nm)	Bias Voltage (V)	Responsivity (AW^−1^)	Reference
WS_2_ nanoflake	Exfoliation	532	1	73	Present work
WS_2_ nanosheets	Hydrothermal intercalation	532	5	4 × 10^−3^	[19]
WS_2_ films	PLD	635	9	0.51	[26]
WS_2_ nanofilm	Sputtering	365	5	53.3	[52]
WS_2_ monolayer	CVD	532	10	0.52 × 10^−3^	[56]
WS_2_ monolayer	CVD	500	1	7.3	[69]
WS_2_ multilayer	CVD	458–647	5	92 × 10^−6^	[70]

## Data Availability

The data presented in this study are available on request from the corresponding author.
